# Manual polishing of 3D printed metals produced by laser powder bed fusion reduces biofilm formation

**DOI:** 10.1371/journal.pone.0212995

**Published:** 2019-02-27

**Authors:** Marissa McGaffey, Alex zur Linden, Nathanael Bachynski, Michelle Oblak, Fiona James, J. Scott Weese

**Affiliations:** 1 Department of Clinical Studies, Ontario Veterinary College, University of Guelph, Guelph, ON, Canada; 2 Department of Pathobiology, Ontario Veterinary College, University of Guelph, Guelph, ON, Canada; University of Vigo, SPAIN

## Abstract

Certain 3D printed metals and surface finishes may be better suited for canine patient specific orthopedic implants on the basis of minimizing potential bacterial biofilm growth. Thirty disks each of titanium alloy, stainless steel, and cobalt chromium alloy were 3D printed via laser powder bed fusion. Fifteen disks of each metal were subsequently polished. After incubation with a robust biofilm-forming methicillin-resistant *Staphylococcus pseudintermedius* isolate, disks were rinsed and sonicated to collect biofilm bacteria. Serial dilutions were plated on blood agar, and colony forming units were counted log (ln) transformed for analysis of variance. Interference microscopy quantified surface roughness for comparison to biofilm growth. Scanning electron microscopy on both pre- and post-sonicated disks confirmed biofilm presence and subsequent removal, and visualized surface features on cleaned disks. Significantly more bacteria grew on rough versus polished metal preparations (p < 0.0001). Titanium alloy had more bacterial biofilm growth compared to cobalt chromium alloy (p = 0.0001) and stainless steel (p < 0.0001). There were no significant growth differences between cobalt chromium alloy and stainless steel (p = 0.4737). Relationships between biofilm growth and surface roughness varied: positive with the rough preparations and negative with the smooth. Polished preparations had increased variance in surface roughness compared to rough preparations, and within disk variance predominated over between disk variance for all preparations with the exception of rough cobalt chromium alloy and rough stainless steel. Using scanning electron microscopy, bacterial biofilms tended to form in crevices. Overall, manual polishing of 3D printed surfaces significantly reduced biofilm growth, with preparation-specific relationships between surface roughness and biofilm growth. These results suggest that metallic implants produced by laser powder bed fusion should be polished. Further research will elucidate the optimal surface roughness per preparation to reduce potential biofilm formation and implant associated infection.

## Introduction

Postoperative surgical site infections (SSIs) are a common and potentially devastating complication in human and veterinary patients and are estimated to occur in 2–5% of human surgical patients in the United States [1,2]. In veterinary medicine, the incidence of SSIs vary between populations, with reported ranges of 0.8–18.1% [3–7]. SSIs come with a high cost to both patient and client as a result of increased medication and revision surgery requirements, extended hospital stays, financial expenditures, and patient mortality [1,4,6,7,8]. Medical advancements have led to an increase in frequency of surgical procedures that include insertion of implants [4], including those made from metals, plastics and ceramics [9]. Further advances have allowed for pre-operative creation of patient-specific implants, which provide direct patient benefits, including reduced time under general anesthesia, reduced need for analgesics, decreased blood loss, reduced risk of infection, decreased need for antibiotics, and improved surgical outcome [9,10], as well as a reduction in intraoperative fluoroscopic navigation, decreasing radiation exposure [10]. The use of 3D printing techniques such as laser powder bed fusion (LPBF) to manufacture detailed patient specific implants is one reason for the increasing use of 3D printing in the medical field [11].

While implants may be beneficial or necessary for some surgical procedures, there is an inherent risk of infection. Surgical procedures involving implants are known to increase the risk of SSIs due to bacterial colonization of the implant surface [4,6,12,13,14] and implant-associated infections can be difficult to treat. In a study by Turk et al., dogs with a medical implant were 5.6 times more likely to develop a SSI than dogs without [13]. For example, canine tibial plateau leveling osteotomy (TPLO) is considered a clean surgical procedure which involves fixation of a metallic implant to the tibia, but has a SSI incidence of 2.5–15.8% [15]. *Staphylococcus* spp., mainly *S*. *pseudintermedius* are the most common isolates from TPLO SSIs [16–18] and from SSIs as a whole [13]. Increasingly, methicillin-resistant *Staphylococcus pseudintermedius* (MRSP) is involved, further complicating treatment.

Methicillin-resistant *Staphylococcus* species are important causes of morbidity and mortality as a result of a variety of opportunistic infections, including SSIs [19]. Methicillin-resistant *Staphylococcus aureus* (MRSA) is a significant cause of morbidity and mortality in hospitalized humans [1,19,20], while both MRSA and MRSP are capable of causing morbidity, mortality and are among the leading causes of SSIs in companion animals [13,19]. Both MRSA and MRSP produce surface-coating biofilms which confer resistance to antibiotics, disinfectants, phagocytosis, and the host immune system, promoting chronic infections such as implant-associated SSIs [21].

With the use and availability of 3D printing technology in human and veterinary medicine [9], there is a need to investigate biofilm forming ability on metals available for 3D printing patient-specific orthopedic implants via LPBF. This study aimed to assess MRSP biofilm growth on different surface finishes of LPBF orthopedic implant metals to help understand the role that surface finish may play in an implant-associated SSI. The implant surface following LPBF is rough due to laser heat diffusion, which causes propagation of powder bonding beyond the area exposed to the laser [22,23]. This phenomenon makes smooth surfaces and corners difficult to manufacture without post-processing modifications [22], and facilitates biofilm adherence [24]. Therefore, tumbling, chemical and electrochemical polishing, blasting, electropolishing, lathe or microabrasive machining, laser polishing, plasma spraying, or manual polishing [25–27] may be employed to smooth the surface [23,28,29,30]. The objective of this study was to assess the role of surface finish, using manual polishing, on biofilm formation of three different LPBF metals. Our hypothesis was that a manually polished finish would result in decreased biofilm formation compared to a rough finish, and our objective was achieved.

## Materials and methods

### Preparation of 3D printed disks and bacterial incubation

**Fig 1:**
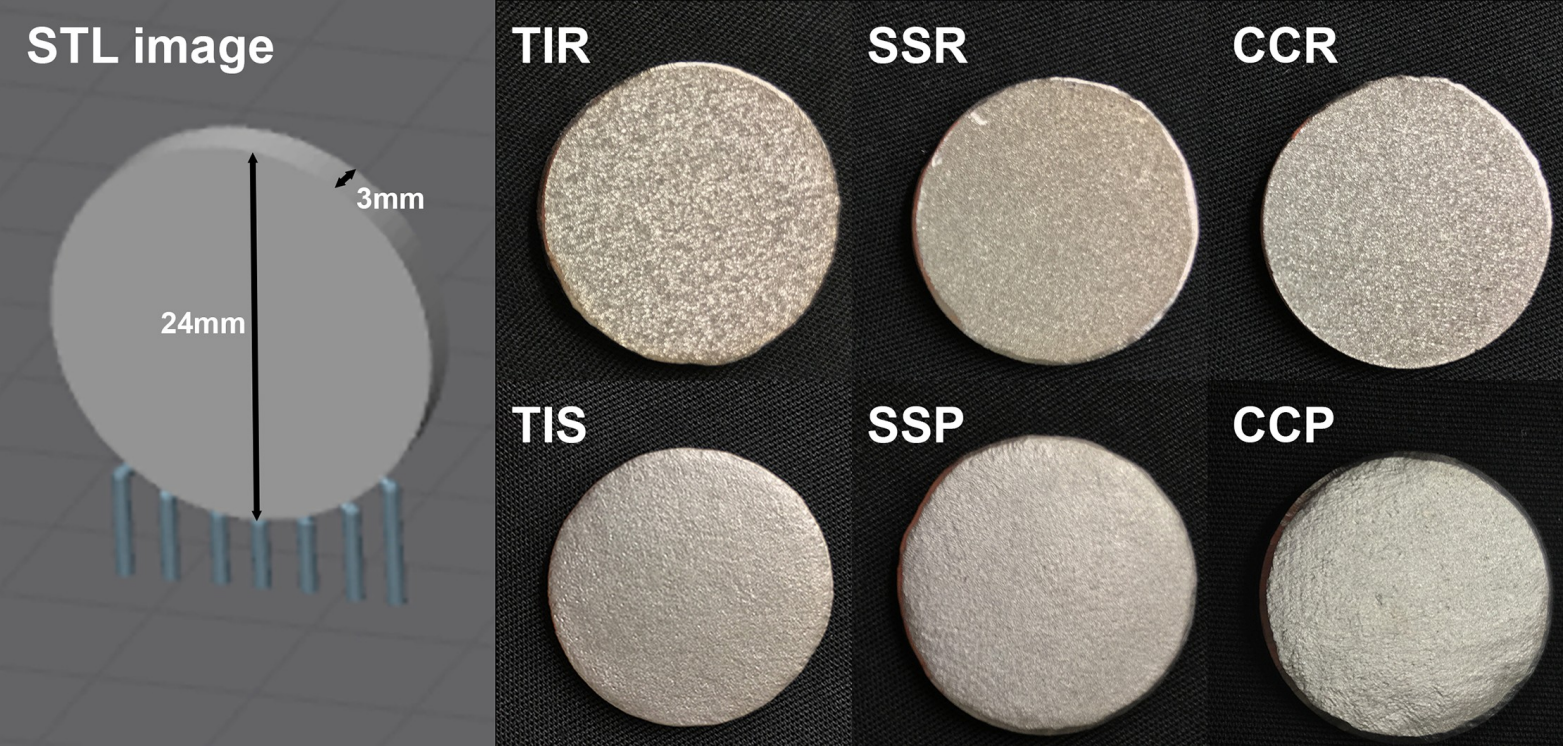
Disk geometry and build orientation (stereolithography (STL) digital image), with photographs of rough and smooth disks. TIR—Titanium alloy rough, TIP—Titanium alloy polished, SSR—Stainless steel rough, SSP—Stainless steel polished, CCR—Cobalt chromium alloy rough, CCP—Cobalt chromium alloy polished.

**Fig 2:**
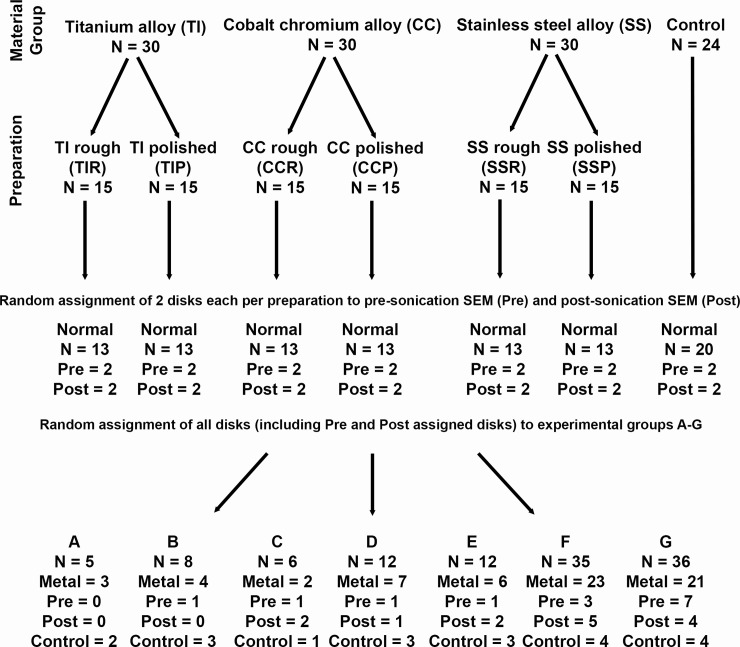
Diagrammatic depiction of disk assortment to experimental groups, with randomization steps indicated.

Ninety disks were 3D printed via LPBF; 30 of each titanium alloy Ti6Al4V (TI), stainless steel 316L (SS) and cobalt chromium alloy CoCr (CC) using standard Renishaw powders (ADEISS, London, ON, CAN; Renishaw plc, Wotton-under-Edge, Gloucestershire, UK). The TI and CC disks were manufactured using a Renishaw AM400 Laser Melting System, and the SS disks were manufactured using a Renishaw AM250 Laser Melting System (Renishaw plc, Wotton-under-Edge, Gloucestershire, UK). Each machine was dedicated to a specific metal type to prevent powder contamination, however all machines contain the same components, materials, and chemicals that interact with the powder, and used a 70 μm laser spot diameter with a 40 μm layer thickness. The disks were 3 mm thick and 24 mm in diameter (S1 Fig), similar to those used in another study [31] but with a vertical build orientation (Fig 1). Each material group was divided into two preparations of 15 disks, one with the original post-LPBF surface unchanged (rough) while the surfaces of the other were polished smooth (polished) using an Espert 500 Ultra-precision brushless micro-grinder with tungsten carbide burs (NSK America Corporation, Hoffman Estates, IL, USA), ranging from 10–40 000 rpm (Fig 1). A random number generator (StatTrek.com) was used to allocate disks to experimental batches (Fig 2). Twenty-four disks were printed via stereolithography (SLA; Formlabs Form 2, Formlabs Inc., Somerville, MA, USA) from black SLA resin (Formlabs Inc., Somerville, MA, USA) at a resolution of 50 micrometres and were included in every experimental batch as indicators of contamination and to flag any outlier groups to ensure bacterial growth was similar across groups (Fig 2). Plastic disks were used as they were significantly less expensive than metal disks. All disks were sterilized using a routine pre-vacuum steam autoclave at 132° C for 4 minutes (Amsco Century P-160H SFPP Steam Sterilizer, STERIS Corporation, Mentor, OH, USA).

One MRSP isolate (MRSP A12), a sequence type 111 isolate that was previously shown to be a robust biofilm producer [32] was used. A pure 24 hour growth of this isolate was suspended in 10 mL of tryptone soy broth with 1% glucose (TSBG) to achieve a 0.5 McFarlane standard solution. Disks were placed in sterile containers with 10 mL of the TSBG-MRSP solution. Following a 24 hour static incubation at 37°C, disks were rinsed 3 times with 10 mL of phosphate buffered saline (PBS) to remove planktonic bacteria. Two of the fifteen disks in each preparation were randomly chosen for pre-sonication scanning electron microscopy (SEM; FEI Quanta FEG 250 SEM, Thermo Fisher Scientific, Hillsboro, OR, USA). The thirteen remaining rinsed disks were transferred to a new sterile container with 10 mL TSBG and sonicated (Branson 2510MT Ultrasonic Cleaner, Branson Ultrasonics Corporation; Danbury, CT, USA) for 5 minutes to remove biofilm-associated bacteria. Following sonication, two disks from each preparation were randomly chosen for post-sonication SEM. Serial dilutions of the TSBG-biofilm solution were performed from a minimum of 10^−4^ to a maximum of 10^−7^, with 100 ul aliquots inoculated onto blood agar and incubated for 24 hours. Colony forming units (CFU) were manually counted after incubation and plates with 30–300 CFU were used to calculate the mean CFU/disk for each group.

### Surface characterization by scanning electron microscopy and surface profilometry

Two disks per preparation underwent fixation post-rinsing and pre-sonication, and 2 disks were fixed post-sonication after being used to calculate CFU/disk. Fixation was carried out via addition of 10 mL of a 2% glutaraldehyde solution to each disk for a minimum of 2 days at 4°C. After disks were rinsed with PBS, 4 drops of osmium tetroxide were placed onto the surface and allowed to sit for 15 minutes. Water was used to rinse the disks twice to remove any residual osmium. A gold sputter coating was applied using a Denton Desk V TSC Sputter Coater (Denton Vacuum, Moorestown, NJ, USA) and subsequent SEM was carried out with representative images taken at magnifications of 100x, 400x, 3000x, and 6000x for each disk.

All remaining disks were gently cleaned using an enzymatic cleaner (Asepti-Zyme, Ecolab, St. Paul, MN, USA) and a soft bristle toothbrush to remove all surface debris. One disk from each preparation was sputter coated as above and SEM images were taken to evaluate the surface features at 100x, 400x, 3000x, and 6000x magnifications. The remainder of the disks and the uncoated underside of the SEM fixed disks underwent surface profilometry via interference microscopy using a Wyko NT3300 Optical 3D Profiling System (Veeco, Plainview, NY, USA) to obtain Ra (average absolute profile height deviation from the mean line over the evaluation length) and Rq (the root mean square average of the profile heights over the evaluation length) measurements for quantifying surface roughness.

### Statistical analysis

To explore biofilm growth per preparation, mean CFU/disk for each material group were natural log (ln) transformed. A one-way ANOVA was used to compare across preparations and assess significance among the SLA disks, with the categorical variable being preparation.

A one-way analysis of covariance was performed with preparation as the categorical variable and Ra and Rq values as the explanatory variables to determine the effect of surface roughness on CFU. Interactions and quadratics for preparations in the model were attempted. A one-way ANOVA with subsampling and disk as the categorical variable was used to compare Ra and Rq within disks. The Spearman correlation was used to assess the correlation between Ra and Rq.

To assess ANOVA assumptions residual analyses were conducted, including testing formally for normality using the Shapiro-Wilk, Kolmogorov-Smirnov, Cramer-von Mises, and Anderson-Darling tests. The Akaike information criterion and Bartlett’s test were used to check for unequal variance, and an exact p value was computed to confirm Bartlett’s test. In addition, residuals were plotted against the predicted values and explanatory variables used in the model to identify outliers and unequal variance. Unequal variance was allowed for in the model using the PROC MIXED statement. Results were reported as means with 95% confidence intervals. Pairwise comparisons and contrasts between material groups were carried out to determine significant differences and biofilm growth ratios. The Intraclass Correlation Coefficient (ICC) was used to determine the variance attributable to between versus within disk variance (Eq 1). P values for differences among groups were adjusted for multiple comparisons using Tukey’s method (adjp) and values of ≤ 0.05 were considered significant. Non-adjusted values of p ≤ 0.05 were considered significant for contrast statements. Statistical software (SAS 9.4, Cary, NC, USA) was used to perform all statistical analyses and all results were reported following ln transformation.

$$ICC=\frac{\sigma_{\alpha }^{2}}{\sigma_{\alpha }^{2}+\sigma_{\varepsilon }^{2}}$$ Eq 1

## Results

### Biofilm growth

**Fig 3:**
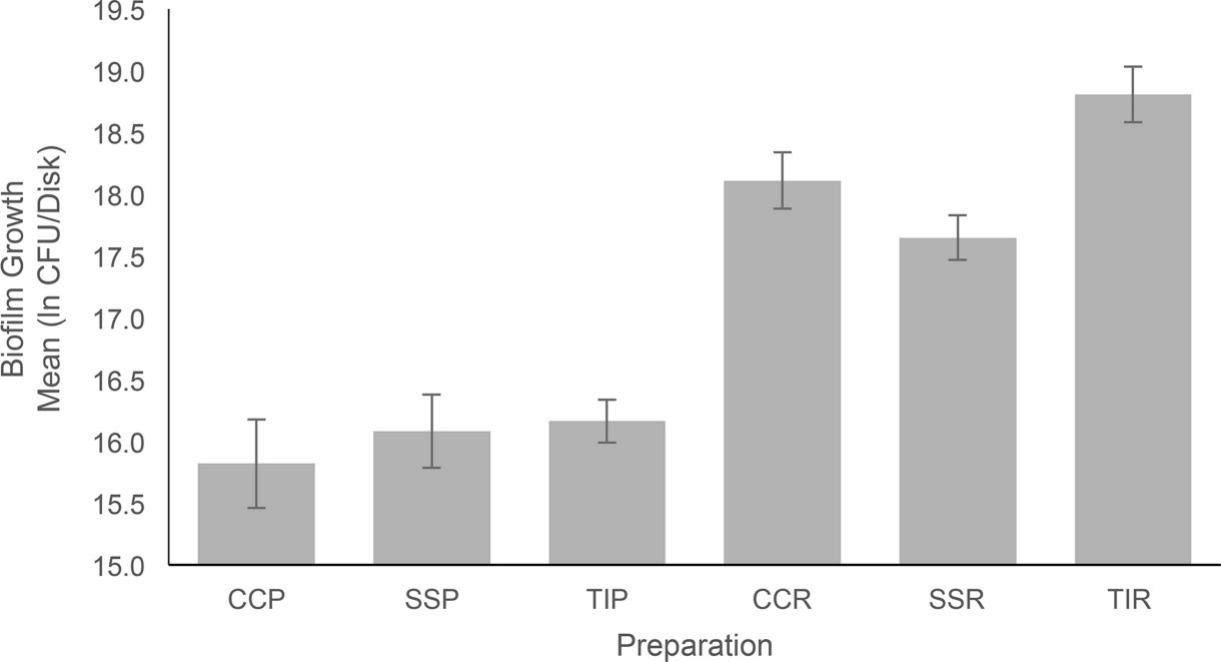
Graph of biofilm growth versus preparation, via mean natural log (ln) transformed CFU/disk per preparation, with 95% confidence intervals demarcated. CCP—Cobalt chromium alloy polished, SSP—Stainless steel polished, TIP—Titanium alloy polished, CCR—Cobalt chromium alloy rough, SSR—Stainless steel rough, TIR—Titanium alloy rough.

There were no significant differences in biofilm growth between the control SLA disks. The maximum and minimum mean biofilm growth per preparation returned after natural log (ln) transformation of CFU/disk were 18.8 (TIR) and 15.8 (CCP), respectively (Fig 3).

Individual comparisons of rough vs. polished preparations showed that TIR, CCR, and SSR grew significantly more biofilm than TIP, CCP, and SSP respectively (p < 0.0001; Fig 3). There were no significant differences in biofilm growth among polished preparations (Table 1).

**Table 1:** Pairwise comparisons of biofilm growth between all preparations types, based on mean natural log (ln) transformed CFU/disk values.

*Preparations Compared*	*Ratio*	*p Value (Adjusted for multiple comparisons)*	*Lower Limit (Adjusted for multiple comparisons)*	*Upper Limit (Adjusted for multiple comparisons)*
CCP vs. CCR	0.10112	<0.0001*	0.05307	0.1926
CCP vs. SSP	0.76848	0.9194	0.37852	1.5602
CCP vs. SSR	0.16052	<0.0001*	0.08726	0.2953
CCP vs. TIP	0.70789	0.6035	0.38627	1.2973
CCP vs. TIR	0.05027	<0.0001*	0.02641	0.0957
CCR vs. SSP	7.60001	<0.0001*	4.30054	13.4309
CCR vs. SSR	1.58749	0.0336*	1.02163	2.4668
CCR vs. TIP	7.00081	<0.0001*	4.52889	10.8219
CCR vs. TIR	0.49718	0.0008*	0.30544	0.8093
SSP vs. SSR	0.20888	<0.0001*	0.12303	0.3546
SSP vs. TIP	0.92116	0.9991	0.54490	1.5572
SSP vs. TIR	0.06542	<0.0001*	0.03705	0.1155
SSR vs. TIP	4.40997	<0.0001*	3.01064	6.4597
SSR vs. TIR	0.31319	<0.0001*	0.20176	0.4862
TIP vs. TIR	0.07102	<0.0001*	0.04599	0.1097

Ratios are of medians (ie. back transformations), which are reported in CFU/disk. CCR—cobalt chromium alloy rough, CCP—cobalt chromium alloy polished, SSR—stainless steel rough, SSP—stainless steel polished, TIR—titanium alloy rough, TIP—titanium alloy polished.

* Denotes statistically significant differences (p ≤ 0.05).

Group contrasts of rough vs. polished metals, and among SS vs. TI, and CC vs. TI material groups returned significant differences, with rough metals growing more biofilm than polished, and TI growing more than either SS or CC (Table 2). CC vs. SS were not significantly different with regard to biofilm growth (Table 2).

**Table 2:** Summary of group contrast statements performed returning estimated differences between mean natural log (ln) transformed CFU/disk for each preparation type, including rough vs. polished preparations and between all three material types, with p values and 95% confidence intervals.

*Preparation or Material Groups Compared*	*Estimate*	*p Value*	*Lower Limit*	*Upper Limit*
Polished vs. Rough	-2.1674	<0.0001*	-2.3739	-1.9610
Stainless steel vs. titanium alloy	-0.6215	<0.0001*	-0.8470	-0.3961
Cobalt chromium alloy vs. titanium alloy	-0.5221	0.0001*	-0.7781	-0.2662
Cobalt chromium alloy vs. stainless steel	0.0994	0.4737	-0.1752	0.3740

* Denotes significant differences (p ≤ 0.05).

Variances in biofilm growth per preparation appeared unequal when residuals were plotted against predicted values. This was confirmed by the Akaike information criterion and Bartlett’s test, and then accounted for in the model. Cobalt chromium alloy polished (CCP) displayed the most variance in biofilm growth while stainless steel alloy rough (SSR) displayed the least, with σ^2^ estimates of 0.4227 and 0.0907 respectively.

### Surface roughness versus biofilm growth

**Fig 4:**
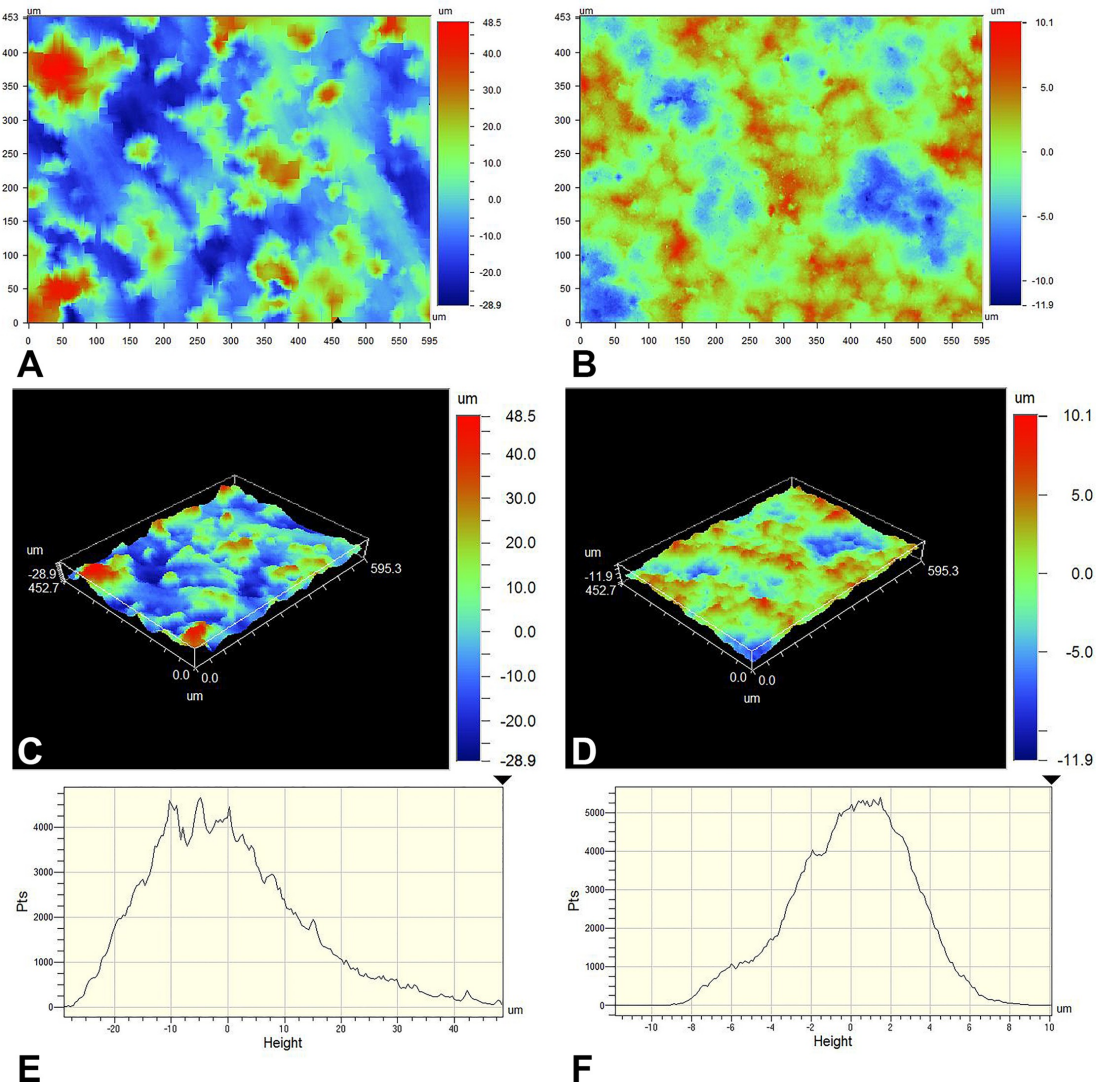
**Surface profilometry contour plots (A,B), 3D plots (C,D), and histogram analyses (E,F) of rough (A,C,E) and polished (B,D,F) stainless steel disks.** A-D depict positive (red) and negative (blue) surface deviations from the mean (green). E-F represent the number of sampled points per surface height.

**Fig 5:**
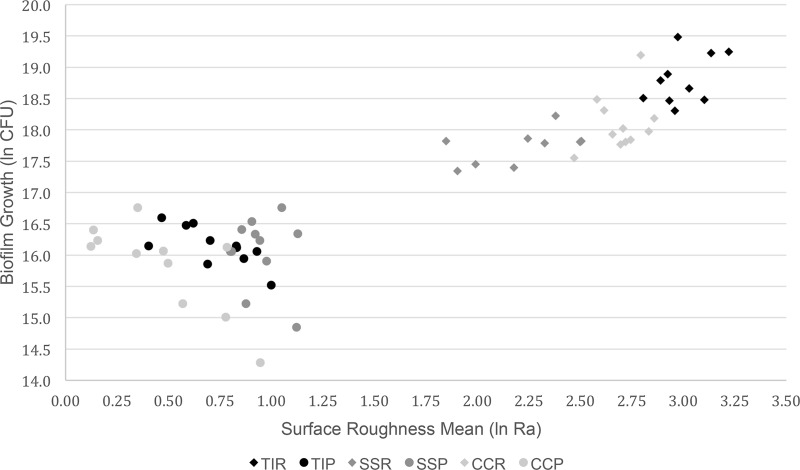
Biofilm growth based on natural log (ln) transformed CFU/disk versus mean natural log (ln) transformed surface roughness (Ra) by disk. TIR—Titanium alloy rough, TIP—Titanium alloy polished, SSR—Stainless steel rough, SSP—Stainless steel polished, CCR—Cobalt chromium alloy rough, CCP—Cobalt chromium alloy polished.

A simple interaction between disks was found, and quadratics for preparation were non-significant. Ra and Rq were found to be very highly correlated under the Spearman Correlation with a coefficient of 0.99917, so Ra was used to represent surface roughness and for all biofilm growth vs. roughness analyses. Mean surface roughness (Ra) ranged from 9.1743 μm to 20.3140 μm among the rough preparations and 1.7253 μm to 2.6159 μm among the smooth preparations (Table 3; Fig 4). Plotting mean ln CFU per disk versus mean Ra per disk confirmed smooth disks grew less biofilm than rough, but displayed relationships by preparation (Fig 5).

**Table 3:** Summary of the mean surface area per preparation with upper and lower limits, equations for the relationship between biofilm growth and surface area per preparation, and the p value returned for the significance of that relationship.

*Preparation*	*Mean Ra (μm)*	*Lower Limit*	*Upper Limit*	*Equation for Slope of Biofilm Growth vs*. *Ra per Preparation*	*p Value*
TIR	20.3140	18.1448	22.1514	log(*TIR*) = 14.6346 + 1.3923*x*	0.2262
TIP	2.1322	2.0757	2.3253	log(*TIP*) = 16.9696 − 1.1443*x*	0.1127
SSR	9.1743	8.8252	9.7678	log(*SSR*) = 16.3673 + 0.6141*x*	0.3189
SSP	2.6159	2.5750	2.8587	log(*SSP*) = 16.7328 − 0.7087*x*	0.5487
CCR	15.0034	14.8362	16.3371	log(*CCR*) = 14.9508 + 1.1667*x*	0.3287
CCP	1.7253	1.6294	1.8710	log(*CCP*) = 16.7265 − 1.9095*x*	0.0002*

* Denotes significant differences (p ≤ 0.05).

Ra—surface roughness, TIR—titanium alloy rough, TIP—titanium alloy polished, SSR—stainless steel rough, SSP—stainless steel polished, CCR—cobalt chromium alloy rough, CCP—cobalt chromium alloy polished.

The relationship between biofilm growth and Ra was only significantly correlated for the CCP preparation (Table 3). The lack of significant correlations between Ra and biofilm growth for the remaining preparations is suspected to be a result of insufficient sample size. All relationships for Ra versus biofilm growth were positive for the rough preparations, and negative for the polished preparations (Table 3, Fig 5). Comparing the slopes of the Ra versus biofilm growth relationships showed significant differences between the slopes of CCR and CCP (p = 0.0196), CCP and SSR (p = 0.002), and CCP and TIR (p = 0.01). Combining the data from all smooth preparations and comparing its Ra versus biofilm growth relationship to that of all rough preparations also returned a significant difference in the two slopes (p = 0.0036).

Covariance parameter estimates of roughness within disks were all significant, with Ra variance within a preparation being CCR < SSR < TIR < SSP < TIP < CCP (Table 4). The between disk variance predominated for CCR, while within disk variance predominated for all other preparations (Table 4). Variance as a result of between disk differences, versus variance as a result of within disk differences was equal to 14.88%, 27.71%, 38.96%, 46.87%, 56.01% and 57.78% for CCP, TIP, SSP, TIR, SSR and CCR, respectively.

**Table 4:** Summary of estimates for variance in surface roughness (Ra) for each material group preparation.

*Preparation*	*Variance of Ra* *(Estimate of σ*^*2*^*)*	*p Value*	*Lower Limit*	*Upper Limit*
CCR	0.01557	<0.0001*	0.01016	0.02683
SSR	0.01674	0.0002*	0.01039	0.03141
TIR	0.02416	<0.0001*	0.01607	0.04038
SSP	0.03339	<0.0001*	0.02210	0.05625
TIP	0.05558	<0.0001*	0.03664	0.09427
CCP	0.1219	<0.0001*	0.07953	0.2100

* Denotes significant differences (p ≤ 0.05).

### Scanning electron microscopy

**Fig 6:**
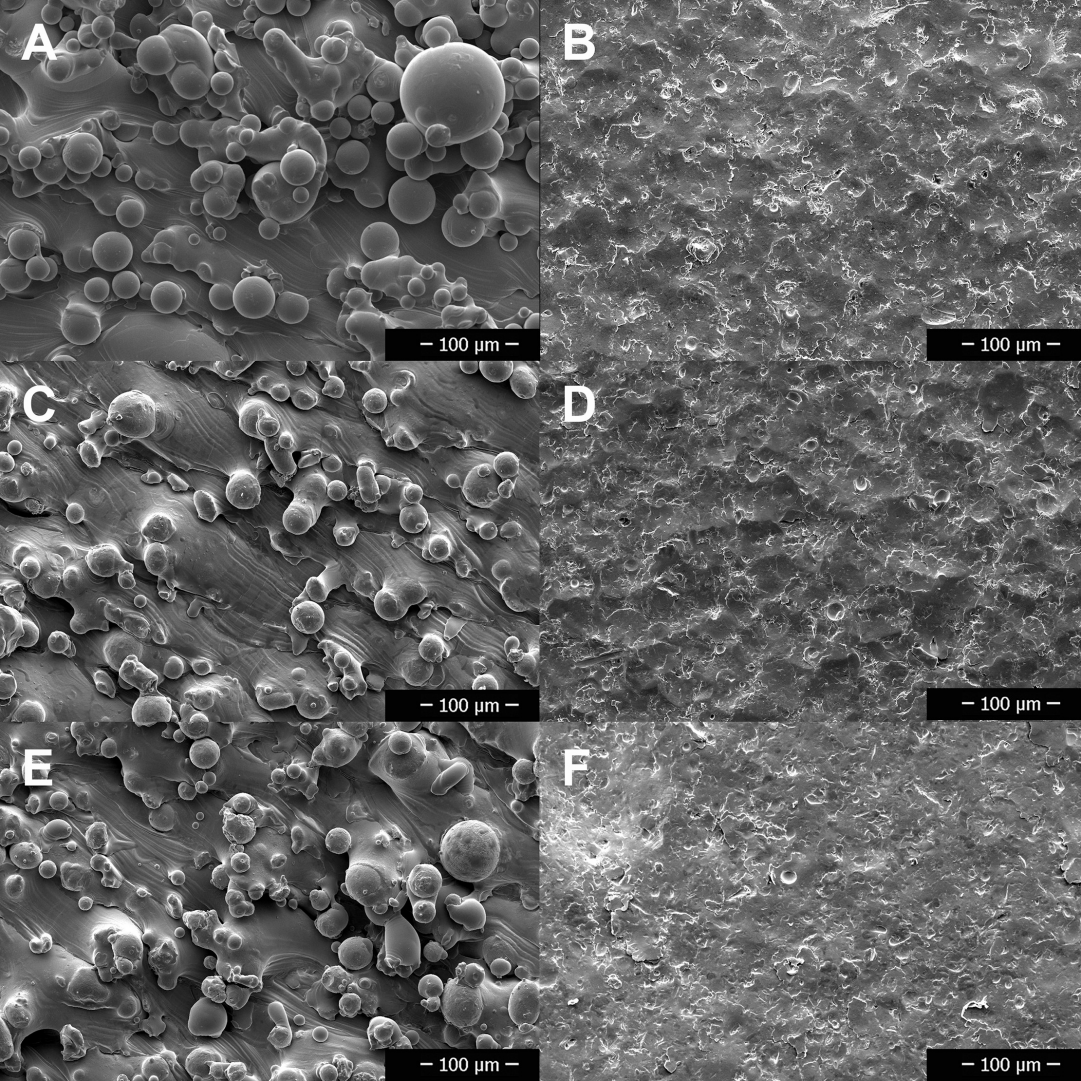
Scanning electron microscope images at 400x magnification of the surfaces of the rough and smooth (polished) forms of the three different metal disks. A—Titanium alloy rough, B—Titanium alloy polished, C—Stainless steel rough, D—Stainless steel polished, E—Cobalt chromium alloy rough, F—Cobalt chromium alloy polished.

SEM images of rough and polished disks (Fig 6) demonstrated differences in microscopic surface features. Visual comparison and interpretation of SEM images at equal magnifications confirmed subjectively that rough metals had increased surface roughness compared to polished metals (Fig 6). Subjectively, the surface features were more prominent on TIR and least prominent on SSR, which was supported by the objective Ra measurements presented in Table 3.

**Fig 7:**
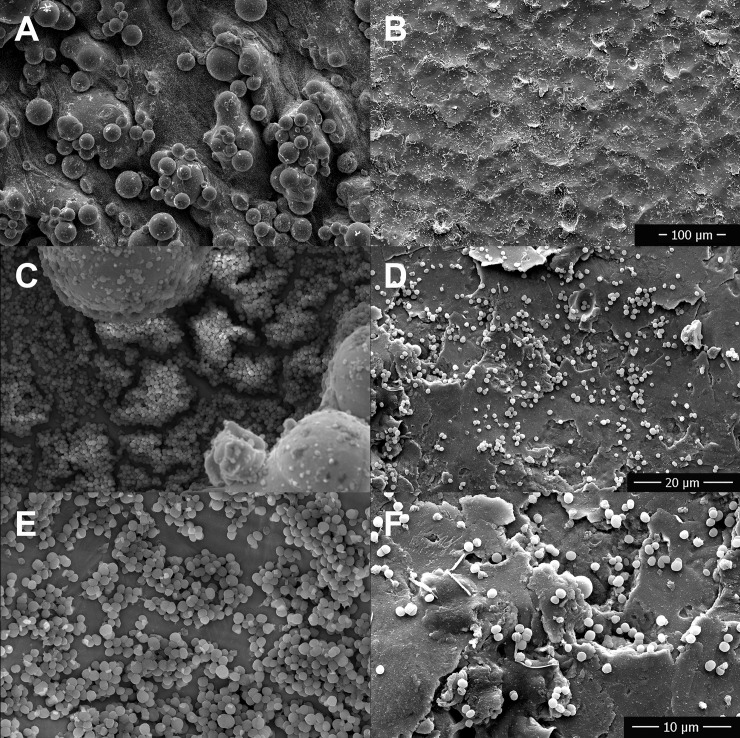
**Comparison of biofilm growth between 3D printed titanium alloy disks with a rough (A, C, E) versus a polished finish (B, D, F) using scanning electron microscopy.** Titanium alloy disks were imaged following 24 hours incubation in bacterial culture and after rinsing to remove planktonic bacteria. A,B 400 x magnification, C,D 3000 x magnification, E,F 6000 x magnification.

**Fig 8:**
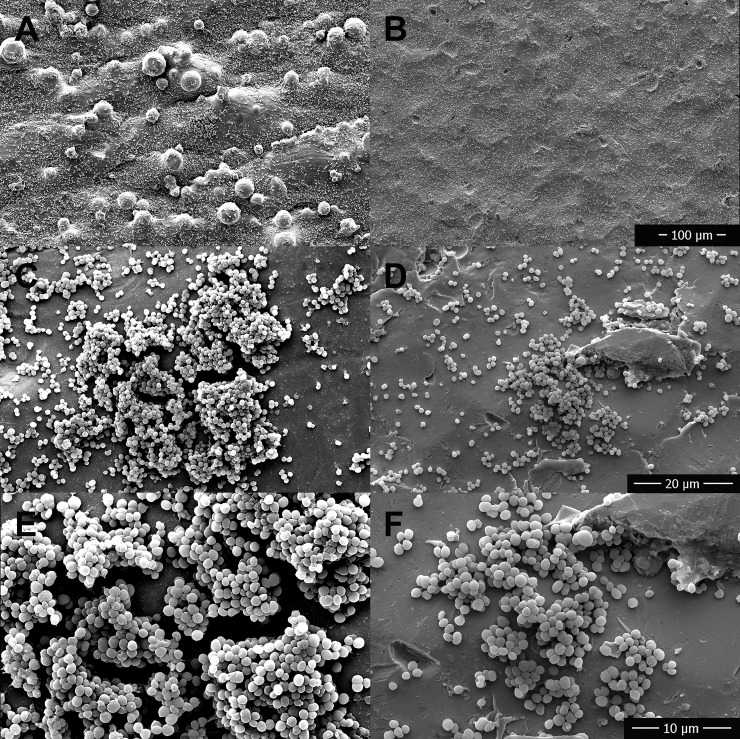
**Comparison of biofilm growth between 3D printed stainless steel disks with a rough (A, C, E) versus a polished finish (B, D, F) using scanning electron microscopy.** Stainless steel disks were imaged following 24 hours incubation in bacterial culture and after rinsing to remove planktonic bacteria. A,B 400 x magnification, C,D 3000 x magnification, E,F 6000 x magnification.

**Fig 9:**
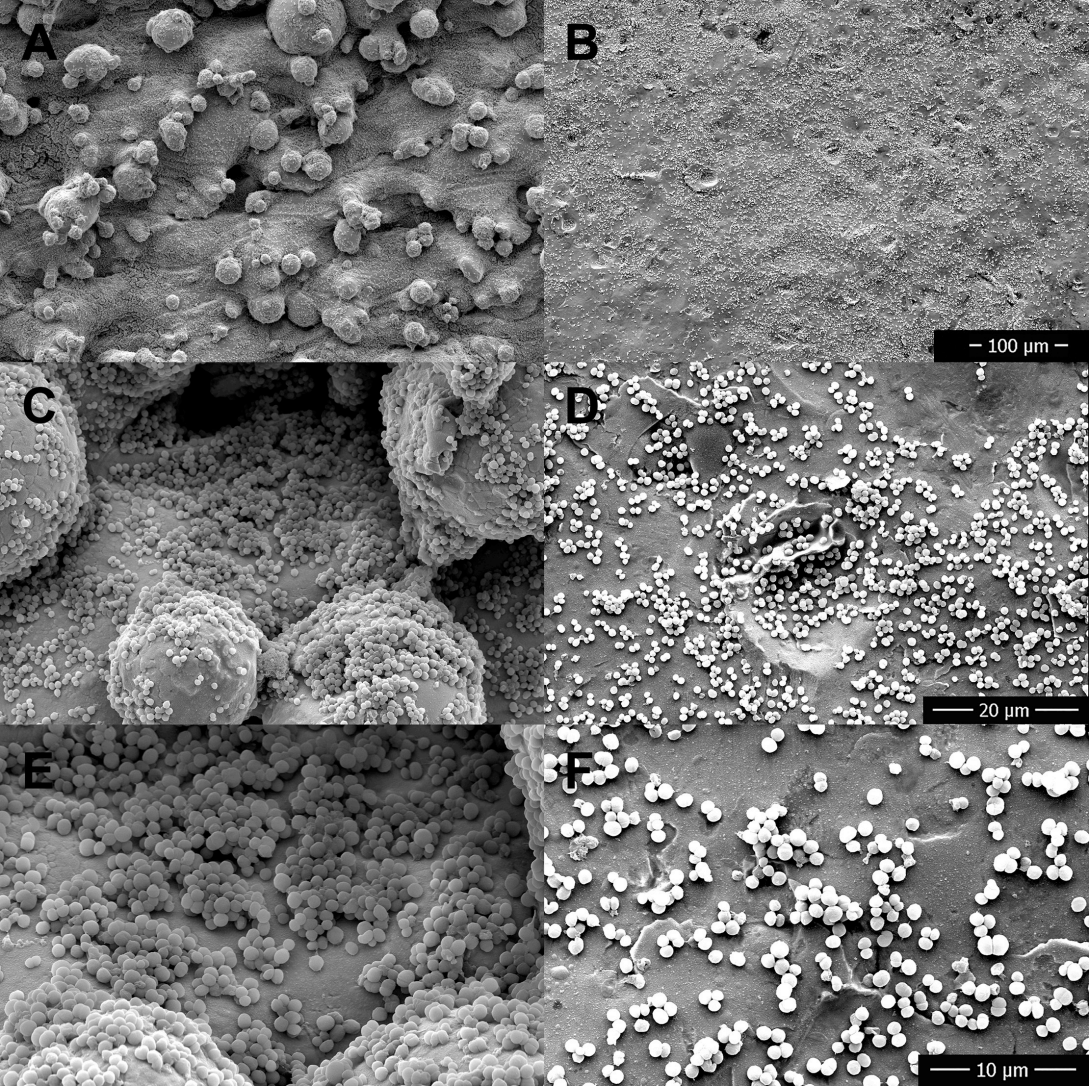
**Comparison of biofilm growth between 3D printed cobalt chromium alloy disks with a rough (A, C, E) versus a polished finish (B, D, F) using scanning electron microscopy.** Cobalt chromium alloy disks were imaged following 24 hours incubation in bacterial culture and after rinsing to remove planktonic bacteria. A,B 400 x magnification, C,D 3000 x magnification, E,F 6000 x magnification.

Visual comparison and interpretation of SEM images obtained after incubation with bacteria and saline flushing found subjectively increased bacterial biofilm growth on rough surfaces versus polished surfaces for TI (Fig 7), SS (Fig 8), and CC (Fig 9). Bacteria were found subjectively in greater quantities in crevices, which appeared more frequently on the rough disks than the smooth disks. SEM images obtained of disks following removal of adhered biofilm bacteria via sonication identified some persistent bacteria on the disks in crevices and sheltered areas, but the majority had been removed with no obvious clumps of bacteria.

## Discussion

### Biofilm formation and surface roughness

The lack of significant differences in CFU/disk between the control SLA disks indicated there were no experimental batches with outlying biofilm formation or bacterial growth. MRSP biofilm growth was significantly reduced in all polished preparations relative to rough preparations, with no significant differences among polished preparations. Surface roughness (Ra) positively influenced biofilm growth within rough preparations and negatively in smooth preparations, though only the slopes of the CCR and CCP preparations were determined to be significantly different, with the available sample size. CCP was the only preparation that returned a significant relationship between Ra and biofilm formation (p = 0.0002). Surfaces above Ra = 0.2 μm have been reported to facilitate biofilm growth, leading 0.2 μm to be suggested as the minimal biofilm-forming threshold for surface roughness [33–35]. None of the polished preparations had a mean Ra of ≤0.2 μm and all had biofilm growth. The negative relationship seen between Ra and biofilm growth for the polished preparations is unusual and cannot be explained. This could perhaps be a type I sampling error, and increasing the sample size may aid in elucidating the relationships between Ra and biofilm formation for manually polished metal disks.

The positive relationships seen between Ra and biofilm growth for the rough preparations supports findings in the literature that rough surfaces facilitate increased bacterial biofilm growth through increasing the surface area for adhesion and providing protection against shear forces during initial adhesion [34–36]. This was supported in this study by the subjective finding that biofilms were found on SEM images more frequently in cracks and protected areas. Additionally, Wu et al. found that a species of *Streptococcus* preferred concave features such as valleys, depressions and pits, all of which function to enhance bacteria-surface area contact [37]. *Staphylococcus* shares many features with *Streptococcus*, therefore it seems reasonable to assume that they would have similar predispositions for preferential biofilm formation in specific locations or as a result of specific surface features. For example, both are pathogenic, biofilm-forming opportunistic bacteria [38,39]. Of their subspecies, *Staphylococcus aureus* (which is very similar in morphology and behaviour to MRSP) and *Streptococcus pyogenes* are cocci, with diameters of 0.7–0.9 μm and 0.7 μm, respectively [38].

The results of this in vitro study suggest manual polishing of implants may significantly reduce biofilm growth in vivo compared to implants with their surface left unchanged post LPBF. An optimal Ra may exist at which minimal biofilm growth is observed for each material group, however, this was not determined in this study. Manual polishing in our study did result in significantly reduced surface roughness as measured by Ra values, CFU/disk, and subjectively observed with SEM, however, smoother surfaces could improve on these findings.

It has been established that osseointegration of titanium implants is facilitated through increasing the surface area of the implant, either through surface texturing [26,40,41] or the inclusion of perforations through the implant [40]. Osteoblasts attach more rapidly to rougher surfaces, and increasing porosity increases osteoblast growth, nutrient delivery and waste removal [42]. Furthermore, inclusion of interconnected pores increases osteoblast migration and colonization of the whole structure [43]. In fact, smooth titanium implants have a lower bone-implant contact and are more easily detached than textured implants [44]. This is ideal for implants that may require later removal such as those used in trauma or pediatric patients and may reduce morbidity related to implant removal [45], but has implications for both currently used metallic implants and future manufacturing methods where a tradeoff between osseointegration and an implant’s predisposition for biofilm colonization should be taken into account.

It was hypothesized that differences in surface roughness among SSR, TIR and CCR would be attributable to differences in the characteristics of the metal powders, namely their thermal conductivity. Thermal conductivity is positively related to homogeneity of the melt pool and is temperature dependent [46,47]. If the volume energy density (J/mm^2^) imparted by the laser (a function of scanning speed and laser power, among others) into the metal powder is too low the melt pool size decreases and partial melting results [48–49]. Conversely, increasing laser energy past an optimal point results in adherence of adjacent particles to the melt pool, which creates a void around the area [49]. Partial melting, adherence of adjacent particles, and the creation of voids from which the adjacent particles arose all function to increase surface roughness (Ra). With information obtained from the supplier regarding the metal powders’ thermal conductivity, it was determined that there is an inverse relationship between thermal conductivity and surface roughness of the unaltered metal disks, with increasing thermal conductivity decreasing the surface roughness. Thermal conductivity for the titanium alloy is 6–8 W/mK with a mean surface of roughness of 20.314 μm, for cobalt chrome is 13 W/mK with a surface roughness of 15.0034 μm, and for stainless steel is 16.2 W/mK with a surface roughness of 9.1743 μm. This could also be visualized in the SEM images of the unaltered (rough) preparations (Figs 5–8). This suggests that metals with lower thermal conductivity produce rougher surfaces via LPBF. An implication of this finding is such that future implant material development may include an evaluation of the thermal conductivity of the metal powder in order to facilitate the production of a material with an optimal Ra to reduce biofilm growth.

The variance in surface roughness (Ra) was higher in polished preparations versus rough. The increased variance of Ra among polished preparations may be the result of hand polishing of disks for the smooth preparations, versus production of disks using a uniform particle size, layer thickness and laser diameter, and left without post-processing modifications in the rough preparations. Within a disk, variance was highest in CCP and lowest in CCR, but again lower across rough preparations than smooth preparations. Though manual polishing produced a higher variance in Ra the results did not support that it would be clinically unsuitable since statistically significant biofilm reduction was found for the polished disks. Development and implementation of a surface roughness standard for manual polishing of implants is suggested.

Though nearly limitless surface features can be created through 3D printing techniques, the results of this study suggest that appropriate post-printing polishing is required for 3D printed metallic implants to minimize potential bacterial biofilm growth.

### Limitations

A limitation of this study was that it was performed in vitro, and the results of bacterial biofilm growth in vitro may differ from those found in vivo. Additionally, while bacteria were only grown for 24 hours to create a “biofilm”, in vivo implants would have hours to weeks to months for biofilm growth to occur. This time-frame was selected based on the length of time used in previous studies [24,50] and resulted in biofilm that was detected both by culture and SEM. Misinterpretation of the impact of bacterial growth at 24 hours may have occurred due to the lack of defined SEM criteria for biofilm formation. Only one type of bacteria was tested, and results from other bacteria may differ. A control group made of SS or TI from a commercial implant manufacturing company or made to currently used canine implant specifications would have been ideal to compare the 3D printed disks to, however, one could not be obtained by the authors.

Only the slope of the CCS biofilm growth versus Ra relationship was significant, indicating it was the only group with a sufficient amount of data to correlate the points to the calculated relationship between roughness and biofilm growth (slope). Addition of more disks per preparation would increase the power of the study and potentially provide a clear relationship between biofilm formation and roughness for each disk preparation.

### Conclusions

The results of this study suggest manual polishing of 3D printed metal implants following LPBF may reduce the risk of biofilm formation if an implant associated infection occurs, or at least reduce the chance of biofilm complicating treatment. Manual polishing created more variation in surface roughness, therefore development and implementation of a surface roughness standard for manually polished implants is suggested, or alternatively using an automated polishing method.

## Supporting information

S1 Dataset CFU vs. Disk Data. Biofilm growth (CFU) per disk using ln transform for CFU data (Fig 3, Tables 1 and 2).
(CSV)

S2 Dataset CFU vs. Roughness Data. Biofilm growth (CFU) per disk versus Ra and Rq using ln transform for CFU, Ra & Rq data (Table 3, Fig 5).
(CSV)

S3 Dataset Roughness within Disk Data. Variance in Ra within disk using ln transform for Ra & Rq (Table 4).
(CSV)

S1 Fig STL file of 3D printed disk. (STL)

## References

[1] Engeman JJ, Carmeli Y, Cosgrove SE, Fowler VG, Bronstein MZ, Trivette SL, et al Adverse Clinical and Economic Outcomes Attributable to Methicillin Resistance among Patients with *Staphylococcus aureus* Surgical Site Infection. Clin Infect Dis. 2003;36: 592–598. 10.1086/367653 12594640

[2] Eugster S, Schawalder P, Gaschen F, Boerlin P. A Prospective Study of Postoperative Surgical Site Infections in Dogs and Cats. Vet Surg. 2004;33: 542–550. 10.1111/j.1532-950X.2004.04076.x 15362994

[3] Weese JS. A review of post-operative infections in veterinary orthopaedic surgery. Vet Comp Orthop Traumatol. 2008;21: 99–105. 18545710 10.3415/vcot-07-11-0105

[4] Anderson DJ. Surgical Site Infections. Infect Dis Clin North Am. 2011;25: 135–153. 10.1016/j.idc.2010.11.004 21315998

[5] Kaur S, Harjai K, Chhibber S. *In Vivo* Assessment of Phage and Linezolid Based Implant Coatings for Treatment of Methicillin Resistant *S*. *aureus* (MRSA) Mediated Orthopaedic Device Related Infections. PLoS One. 2016;11: 1–23. 10.1371/journal.pone.0157626 PMC4917197 27333300

[6] Patel A, Calfee RP, Plante M, Fischer SA, Arcand N, Born B. Methicillin-resistant *Staphylococcus aureus* in orthopaedic surgery. Bone Joint J. 2008;90: 1401–1406. 10.1302/0301-620X.90B11.20771 18978255

[7] Gimeno M, Pinczowski P, Vázquez FJ. Porous orthopedic steel implant as an antibiotic eluting device: Prevention of post-surgical infection on an ovine model. Int J Pharm. 2013;452: 166–172. 10.1016/j.ijpharm.2013.04.076 23651643

[8] Verwilghen D, Singh A. Fighting Surgical Site Infections in Small Animals: Are We Getting Anywhere? Vet Clin North Am Small Anim Pract. 2015;45: 243–276. 10.1016/j.cvsm.2014.11.001 25542615

[9] Hoang D, Perrault D, Stevanovic M, Ghiassi A. Surgical applications of three-dimensional printing: a review of the current literature & how to get started. Ann Transl Med. 2016;4: 456–475. 10.21037/atm.2016.12.18 28090512 PMC5220021

[10] Martelli N, Serrano C, van den Brink H, Pineau J, Prognon P, Borget I, et al Advantages and disadvantages of 3-dimensional printing in surgery: A systematic review. Surgery. 2016;159: 1485–1500. 10.1016/j.surg.2015.12.017 26832986

[11] Ventola CL. Medical Applications for 3D Printing: Current and Projected Uses. P T. 2014;39: 704–711. 25336867 PMC4189697

[12] Isackson D, McGill LD, Bachus KN. Percutaneous implants with porous titanium dermal barriers: An *in vivo* evaluation of infection risk. Med Eng Phys. 2011;33: 418–426. 10.1016/j.medengphy.2010.11.007 21145778 PMC3071885

[13] Turk R, Singh A, Weese JS. Prospective Surgical Site Infection Surveillance in Dogs. Vet Surg. 2015;44: 2–8. 10.1111/j.1532-950X.2014.12267.x 25196800

[14] Nelson LL. Surgical Site Infections in Small Animal Surgery. Vet Clin North Am Small Anim Pract. 2011;41: 1041–1056. 10.1016/j.cvsm.2011.05.010 21889700

[15] Nazarali A, Singh A, Moens NM, Gatineau M, Sereda C, Fowler D, et al Association between methicillin-resistant *Staphylococcus pseudintermedius* carriage and the development of surgical site infections following tibial plateau leveling osteotomy in dogs. J Am Vet Med Assoc. 2015;247: 909–916. 10.2460/javma.247.8.909 26421403

[16] Thompson AM, Bergh MS, Wang C, Wells K. Tibial plateau levelling osteotomy implant removal: A retrospective analysis of 129 cases. Vet Comp Orthop Traumatol. 2011;24: 450–456. 10.3415/VCOT-10-12-0172 21975493

[17] Gallagher AD, Mertens WD. Implant Removal Rate from Infection after Tibial Plateau Leveling Osteotomy in Dogs. Vet Surg. 2012;41: 705–711. 10.1111/j.1532-950X.2012.00971.x 22822724

[18] Savicky R, Beale B, Murtaugh R, Swiderski-Hazlett J, Unis M. Outcome following removal of TPLO implants with surgical site infection. Vet Comp Orthop Traumatol. 2013;26: 260–265. 10.3415/VCOT-11-12-0177 23857570

[19] Weese JS, van Duijkeren E. Methicillin-resistant Staphylococcus aureus and Staphylococcus pseudintermedius in veterinary medicine. Vet Microbiol. 2010;140: 418–419. 10.1016/j.vetmic.2009.01.039 19246166

[20] Nixon M, Jackson B, Varghese P, Jenkins D, Taylor G. Methicillin-resistant *Staphylococcus aureus* on orthopaedic wards. Bone Joint J. 2006;88: 812–817. 10.1302/0301-620X.88B6.17544 16720779

[21] Pompilio A, De Nicola S, Crocetta V, Guarnieri S, Savini V, Carretto E, et al New insights in Staphylococcus pseudintermedius pathogenicity: antibiotic-resistant biofilm formation by a human wound-associated strain. BMC Microbiol. 2015;15: 109–122. 10.1186/s12866-015-0449-x 25994406 PMC4440327

[22] Yang S, Leong K, Du Z, Chua C. Review. The Design of Scaffolds for Use in Tissue Engineering. Part II. Rapid Prototyping Techniques. Tissue Eng. 2002;8: 1–11. 10.1089/107632702753503009 11886649

[23] Jung J, Park H, Lee BS, Choi J, Seo B, Kim HK, et al Study on surface shape control of pure Ti fabricated by electron beam melting using electrolytic polishing. Surf Coat Technol. 2017;324: 106–110.

[24] Szymczyk P, Junka A, Ziólkowski G, Smutnicka D, Bartoszewicz M, Chlebus E. The ability of *S*. *aureus* to form biofilm on the Ti-6Al-7Nb scaffolds produced by Selective Laser Melting and subjected to the different types of surface modifications. Acta Bioeng Biomech. 2013;15: 69–76. 23957680

[25] Wysocki B, Idaszek J, Szlązak K, Strzelczyk K, Brynk T, Kurzydlowski KJ, et al Post Processing and Biological Evaluation of the Titanium Scaffolds for Bone Tissue Engineering. Materials. 2015;9: 1–19. 10.3390/ma9030197 PMC5456666 28773323

[26] Longhitano GA, Larosa MA, Munhoz AL, Zavaglia CA, Ierardi MC. Surface Finishes for Ti-6-Al-4V Alloy Produced by Direct Metal Laser Sintering. Mat Res. 2015;18: 838–842.

[27] Sing SL, An J, Yeong WY, Wiria FE. Laser and Electron-Beam Powder-Bed Additive Manufacturing of Metallic Implants: A Review on Processes, Materials and Designs. J Orthop Res. 2016;34: 369–385. 10.1002/jor.23075 26488900

[28] Morrison RJ, Kashlan KN, Flanangan CL, Wright JK, Green GE, Hollister SJ, et al Regulatory Considerations in the Design and Manufacturing of Implantable 3D-Printed Medical Devices. Clin Transl Sci. 2015;8: 594–600. 10.1111/cts.12315 26243449 PMC4626249

[29] Martinez-Marquez D, Mirnajafizadeh A, Carty CP, Stewart RA. Application of quality by design for 3D printed bone prostheses and scaffolds. PLoS One. 2018;13: 1–47. 10.1371/journal.pone.0195291 PMC5896968 29649231

[30] Wang WJ, Yung KC, Choy HS, Xiao TY, Cai ZX. Effects of laser polishing on surface microstructure and corrosion resistance of additive manufactured CoCr alloys. Appl Surf Sci. 2018;443: 167–175.

[31] Sandler N, Salmela I, Fallarero A, Rosling A, Khajeheian M, Kolakovic R, et al Towards fabrication of 3D printed medical devices to prevent biofilm formation. Int J Pharm. 2014;459: 62–64. 10.1016/j.ijpharm.2013.11.001 24239831

[32] Singh A, Walker M, Rousseau J, Weese JS. Characterization of the biofilm forming ability of *Staphylococcus pseudintermedius* from dogs. BMC Vet Res. 2013;9: 93–99. 10.1186/1746-6148-9-93 23641755 PMC3681638

[33] Bollen CM, Lambrechts P, Quirynen M. Comparison of surface roughness of oral hard materials to the threshold surface roughness for bacterial plaque retention: A review of the literature. Dent Mater. 1997;13: 258–269. 11696906 10.1016/s0109-5641(97)80038-3

[34] Teughels W, Van Assche N, Sliepen I, Quirynen M. Effect of material characteristics and/or surface topography on biofilm development. Clin Oral Implants Res. 2006;17: 68–81. 10.1111/j.1600-0501.2006.01353.x 16968383

[35] Yoda I, Koseki H, Tomita M, Shida T, Horiuchi H, Sakoda H, et al Effect of surface roughness of biomaterials on *Staphylococcus epidermidis* adhesion. BMC Microbiology. 2014;14: 234–240. 10.1186/s12866-014-0234-2 25179448 PMC4161769

[36] Jacques M, Aragon V, Tremblay YD. Biofilm formation in bacterial pathogens of veterinary importance. Anim Health Res Rev. 2010;11: 97–121. 10.1017/S1466252310000149 20969814

[37] Wu Y, Zitelli JP, TenHuisen KS, Yu X, Libera MR. Differential response of Staphylococci and osteoblasts to varying titanium surface roughness. Biomaterials. 2011;32: 951–960. 10.1016/j.biomaterials.2010.10.001 20974493

[38] Ann LC, Mahmud S, Bakhori SK, Sirelkhatim A, Mohamad D, Hasan H, et al Antibacterial responses of zinc oxide structures against *Staphylococcus aureus*, *Pseudomonas aeruginosa* and *Streptococcus pyogenes*. Ceram Int. 2014;40: 2993–3001.

[39] Ahmed D, Anwar A, Khan AK, Ahmed A, Shad MR, Khan NA. Size selectivity in antibiofilm activity of 3-(diphenylphosphino)propanoic acid coated gold nanomaterials against Gram-positive *Staphylococcus aureus* and *Streptococcus mutans*. AMB Express. 2017;7: 210–220. 10.1186/s13568-017-0515-x 29164404 PMC5698236

[40] Coathup MJ, Bates P, Cool P, Walker PS, Blumenthal N, Cobb JP, et al Osseo-mechanical induction of extra-cortical plates with reference to their surface properties and geometric designs. Biomaterials. 1999;20: 793–800. 10353662 10.1016/s0142-9612(98)00239-7

[41] Depprich R, Zipprich H, Ommerborn M, Mahn E, Lammers L, Handschel J, et al Osseointegration of zirconia implants: an SEM observation of the bone-implant interface. Head Face Med. 2008;4: 25–32. 10.1186/1746-160X-4-25 18990214 PMC2583968

[42] Haslauer CM, Springer JC, Harrysson OL, Loboa EG, Monteiro-Riviere NA, Marcellin-Little DJ. *In vitro* biocompatibility of titanium alloy discs made using direct metal fabrication. Med Eng Phys. 2010;32: 645–652. 10.1016/j.medengphy.2010.04.003 20447856

[43] Nune KC, Misra RD, Gaytan SM, Murr LE. Interplay between cellular activity and three-dimensional scaffold-cell constructs with different foam structure processed by electron beam melting. J Biomed Mater Res A. 2014;103: 1677–1692 10.1002/jbm.a.35307 25111154

[44] Hulshof FF, Papenburg B, Vasilevich A, Hulsman M, Zhao Y, Levers M, et al Mining for osteogenic surface topographies: In silico design to *in vivo* osseo-integration. Biomaterials. 2017;137: 49–60. 10.1016/j.biomaterials.2017.05.020 28535442

[45] Pearce AI, Pearce SG, Schwieger K, Milz S, Schneider E, Archer CW, et al Effect of Surface Topography on Removal of Cortical Bone Screws in a Novel Sheep Model. J Orthop Res. 2008;26: 1377–1383. 10.1002/jor.20665 18464266

[46] Smith CJ, Tammas-Williams S, Hernandez-Nava E, Todd I. Tailoring the thermal conductivity of the powder bed in Electron Beam Melting (EBM) Additive Manufacturing. Sci Rep. 2017;7: 1–8. 10.1038/s41598-016-0028-x 28874795 PMC5585364

[47] Foteinopoulos P, Papacharalampopoulos A, Stavropoulos P. On thermal modeling of Additive Manufacturing processes. CIRP Journal of Manufacturing Science and Technology. 2018;20: 66–83.

[48] Chen H, Gu D, Xiong J, Xia M. Improving additive manufacturing processability of hard-to-process overhanging structure by selective laser melting. J Mater Process Technol. 2017;250: 99–108.

[49] Yu G, Gu D, Dai D, Xia M, Ma C, Shi Q. On the role of processing parameters in thermal behaviour, surface morphology and accuracy during laser 3D printing of aluminum alloy. J Phys D Appl Phys. 3016;49: 1–15.

[50] Van Hengel IA, Riool M, Fratila-Apachitei LE, Witte-Bouma J, Farrell E, Zadpoor AA, et al Selective laser melting porous metallic implants with immobilized silver nanoparticles kill and prevent biofilm formation by methicillin-resistant *Staphylococccus aureus*. Biomaterials. 2017;140: 1–15. 10.1016/j.biomaterials.2017.02.030 28622569

